# Application of geographic population structure (GPS) algorithm for biogeographical analyses of populations with complex ancestries: a case study of South Asians from 1000 genomes project

**DOI:** 10.1186/s12863-017-0579-2

**Published:** 2017-12-28

**Authors:** Ranajit Das, Priyanka Upadhyai

**Affiliations:** 1 Manipal Centre for Natural Sciences (MCNS), Manipal Academy of Higher Education, Madhav Nagar, Manipal, 576104 Karnataka India; 2 Department of Medical Genetics, Kasturba Medical College, Manipal Academy of Higher Education, Manipal, Karnataka India

**Keywords:** Geographical population structure (GPS), Admixture, Highly admixed populations, Geo-localization, South Asian population history

## Abstract

**Background:**

The utilization of biological data to infer the geographic origins of human populations has been a long standing quest for biologists and anthropologists. Several biogeographical analysis tools have been developed to infer the geographical origins of human populations utilizing genetic data. However due to the inherent complexity of genetic information these approaches are prone to misinterpretations. The Geographic Population Structure (GPS) algorithm is an admixture based tool for biogeographical analyses and has been employed for the geo-localization of various populations worldwide. Here we sought to dissect its sensitivity and accuracy for localizing highly admixed groups. Given the complex history of population dispersal and gene flow in the Indian subcontinent, we have employed the GPS tool to localize five South Asian populations, Punjabi, Gujarati, Tamil, Telugu and Bengali from the 1000 Genomes project, some of whom were recent migrants to USA and UK, using populations from the Indian subcontinent available in Human Genome Diversity Panel (HGDP) and those previously described as reference.

**Results:**

Our findings demonstrate reasonably high accuracy with regards to GPS assignment even for recent migrant populations sampled elsewhere, namely the Tamil, Telugu and Gujarati individuals, where 96%, 87% and 79% of the individuals, respectively, were positioned within 600 km of their native locations. While the absence of appropriate reference populations resulted in moderate-to-low levels of precision in positioning of Punjabi and Bengali genomes.

**Conclusions:**

Our findings reflect that the GPS approach is useful but likely overtly dependent on the relative proportions of admixture in the reference populations for determination of the biogeographical origins of test individuals. We conclude that further modifications are desired to make this approach more suitable for highly admixed individuals.

**Electronic supplementary material:**

The online version of this article (doi: 10.1186/s12863-017-0579-2) contains supplementary material, which is available to authorized users.

## Background

The formulation of appropriate methods to decipher the geographic origins of human populations has been a long-standing quest with biologists and anthropologists. Given that a significant correspondence between geography and genetics has been reflected previously [1, 2], various investigations over the last decade have strived to glean the precise geographic origin of human populations using high-resolution genetic information. The Geographic Population Structure (GPS) algorithm is a recently devised admixture based tool for biogeographical analyses. While GPS has been demonstrated to be superior to other existing methods for tracing the ancestry of human populations [2–7], it may not be accurate for tracing ancestry of recently admixed individuals and groups (up to 1000 years before present) [2, 8]. It relies on extrapolating the genomic similarity between the query and reference populations to infer the likely biogeographical affinity of the former using the geographic locations (latitude and longitude) corresponding to the latter as a reference. GPS has been effectively employed for reconstructing the population history of several populations worldwide [2, 6, 7, 9–11]. However, so far its utility and robustness in accurately localizing highly admixed populations whose genetic structure has been modified by significant demographic, biological and social factors has remained largely unexplored.

India and its neighbouring areas in South Asia are a rich melting-pot of genetic and ethno-linguistic diversity interwoven with unique social practices. Several lines of evidence allude to the presence of prominent signatures of the Late Pleistocene era in Indian population history [12–15]. The demographic landscape of the Indian sub-continent has been modulated by multiple waves of migration during the late glacial maximum (LGM) of Holocene, Neolithic Period, Bronze and Iron Age [16–20]. A long and complex history of admixture between immigrant gene-pools originating primarily in West Eurasia, Southeast Asia [21–27] and the autochthonous Indian lineages [26, 28–30] had generated enormous genetic heterogeneity, which together with the subsequent stringently enforced socio-cultural practices like endogamy [22] gave rise to the distinctive population structure of the Indian sub-continent. Initially it was suggested that extant Indian populations largely arose due to admixture between two ancestral gene-pools, namely Ancestral North Indian (ANI) and Ancestral South Indian (ASI) [31, 32]. However, recent findings support the prevalence of four ancestral genetic components in the mainland Indian populace that included Ancestral Tibeto-Burman (ATB), Ancestral Austro-Asiatic (AAA), in addition to ANI and ASI components [22].

Given the complex history of population dispersal and gene flow in the Indian subcontinent, here we have employed the GPS tool to interrogate publically available whole genome sequence (WGS) data from the Indian sub-continent to ascertain its utility in geo-localization of the corresponding populations. Our dataset included five populations of South Asian ancestry (SAS) available in the 1000 Genomes Project (Phase 3 release), namely two populations originating from the North-Western region of the Indian subcontinent, Gujarati (GIH) and Punjabi (PJL), two populations from the Southern Indian subcontinent,Tamil (STU) and Telugu (ITU), and one population from Eastern region of the Indian subcontinent, Bengalis from Bangladesh (BEB) [33]. Notably three out of the five SAS populations (GIH, STU and ITU) had been sampled from USA and UK. In addition, we assessed data corresponding to eight populations from the North-Western region of the Indian subcontinent, namely Balochi, Brahui, Burusho, Hazara, Kalash, Makrani, Pathan, and Sindhi that are available in the Human Genome Diversity Panel (HGDP) [34, 35] and from 52 Indian groups [31]. We mapped the putative origins of the five SAS populations from the 1000 Genomes project [33] with reference to those available in HGDP [34, 35] and previously published WGS data from Indian populations [31]. We note that GPS geo-localized most genomes including those pertaining to recent migrants from the Indian subcontinent, the GIH, ITU and STU populations with reasonably high accuracy. While likely the lack of appropriate reference populations resulted in moderate-low level of its precision in assigning PJL and BEB genomes. Overall our findings yield a better understanding into the efficacy and limitations of the GPS approach in predicting the biogeographical affiliation and tracing the ancestry of highly admixed populations.

## Methods

### Datasets

The datasets used in the present study comprised of the five South Asian populations available in 1000 Genomes Project, namely Gujarati Indians from Houston, USA (GIH), Punjabis from Lahore, Pakistan (PJL), Indian Telugu from UK (ITU), Sri Lankan Tamil from UK (STU), and Bengalis from Bangladesh (BEB), assessing a total of 489 (103 GIH, 96 PJL, 102 ITU, 102 STU and 86 BEB) samples [33]; eight Pakistani populations (Balochi, Brahui, Burusho, Hazara, Kalash, Makrani, Pathan, and Sindhi) from the Human Genome Diversity Panel (HGDP) dataset 2 (*N* = 197) [34, 35] and 52 previously reported Indian populations (*N* = 378) [31]. File conversions and manipulations were performed using EIG v4.2 [36], VCF tools [37] and PLINK [38]. The VCF files for the Phase 3 release of 1000 Genomes Project were obtained from The International Genome Sample Resource (IGSR) server (http://www.internationalgenome.org/data/). The single nucleotide polymorphisms (SNPs) that passed the default quality control check and were marked as ‘PASS’ in the VCF files were used for further analysis. No additional quality control measures were employed for the HGDP and previously described datasets [31]. All three datasets were made compatible with each other and merged together using PLINK. Overall, 94,759 autosomal SNPs were assessed for 1064 South Asians samples. A separate dataset was generated by including 29 French, 24 Karitiana and 21 Surui samples from Brazil, 19 Melanesians, 17 Papuans, 48 Bedouins from Israel, 51 Palestinians [34, 35], 99 Northern and Western Europeans from Utah, USA (CEU), 103 Han Chinese samples from Beijing, China (CHB), and 108 Yorubans from Ibadan, Nigeria (YRI) [33] to the 1064 South Asian samples (*N* = 1583) for performing a global admixture analysis. A total of 89,727 SNPs were assessed for the global dataset.

### Admixture analysis

The genetic ancestry of all individuals was estimated using an unsupervised clustering algorithm, ADMIXTURE [39]. The optimum number of ancestral components (*K*) was discerned by minimizing the cross-validation error (CVE) [39] implemented in ADMIXTURE v1.3 using a --cv flag to the ADMIXTURE command line. For the global dataset (*N* = 1583), the lowest CVE was estimated for *K* = 13 (Additional file 1: Figure S1), while for the South Asians only dataset (*N* = 1064) the lowest CVE was estimated for *K* = 8 (Additional file 1: Figure S2).

### Biogeographical mapping of south Asian populations

Biogeographical analysis was performed using the Geographic Population Structure (GPS) algorithm [2]. Given a sample of unknown geographic origin and admixture proportions that correspond to putative ancestral populations, the GPS tool converts the genetic distances between the test and the nearest reference populations into geographic distances. Comparing the test samples with the reference populations, GPS finds the geographic coordinates of locations where individuals with similar genotype reside. All supervised admixture proportions were calculated as described previously [40]. Essentially, the GPS algorithm correlates the admixture patterns of individuals of unknown origin with geographical coordinates using the admixture fractions (GEN file) and geographical locations (GEO file) of reference samples.

Prior to applying GPS to elucidate the biogeographical affinity of South Asian populations, we sought to test its accuracy on selected Indian populations corresponding to known latitudes and longitudes. To this end we analyzed 57 individuals from four Indian populations (Brahmin, Kshatriya, Madiga, and Kurumba) described previously [31] and estimated their admixture proportion with respect to eight admixture components corresponding to reference populations.

Subsequently we mapped five South Asian populations (*N* = 489) [33], using previously described Indian [31] and Pakistani populations from HGDP [34, 35] as the reference, interpreting their admixture fractions and geographic locations (latitudinal and longitudinal coordinates). Therefore, the GEN file contains eight admixture coefficients corresponding to 575 individuals across 60 reference populations from around India and Pakistan and the GEO file contains the geographic coordinates (latitude and longitude) for the same.

### Determining the accuracy of GPS prediction

Geographic distances (‘Laws of cosines’, great circle distance) between the physical location of the query samples from 1000 Genomes project and their corresponding GPS assigned locations was calculated using the R package geosphere (https://CRAN.R-project.org/package=geosphere ). For GIH, ITU, and STU genomes that were pertaining to the South Asian diaspora, the capital cities of their ancestral region or country, Ahmedabad (India), Hyderabad, (India) and Colombo (Sri Lanka) respectively were used as their native regional location. Similarly, for PJL and BEB samples the geographic coordinates of Lahore (Pakistan) and Dhaka (Bangladesh) respectively were used for the geographic distance estimation.

## Results

### Clustering of populations

The ancestry of 1064 samples from the South Asians only dataset and 1374 samples from the global dataset was estimated using unsupervised clustering as implemented in ADMIXTURE v1.3 [39]. Model validation by optimum choice of the number of ancestral components (*K*) was achieved for both datasets by minimizing the cross-validation error (CVE). For the global dataset (*N* = 1583), the lowest CVE was estimated for *K* = 13 (Additional file 1: Figure S1), while for the South Asians only dataset (*N* = 1064) the lowest CVE was estimated for *K* = 8 (Additional file 1: Figure S2).

**Fig. 1:**
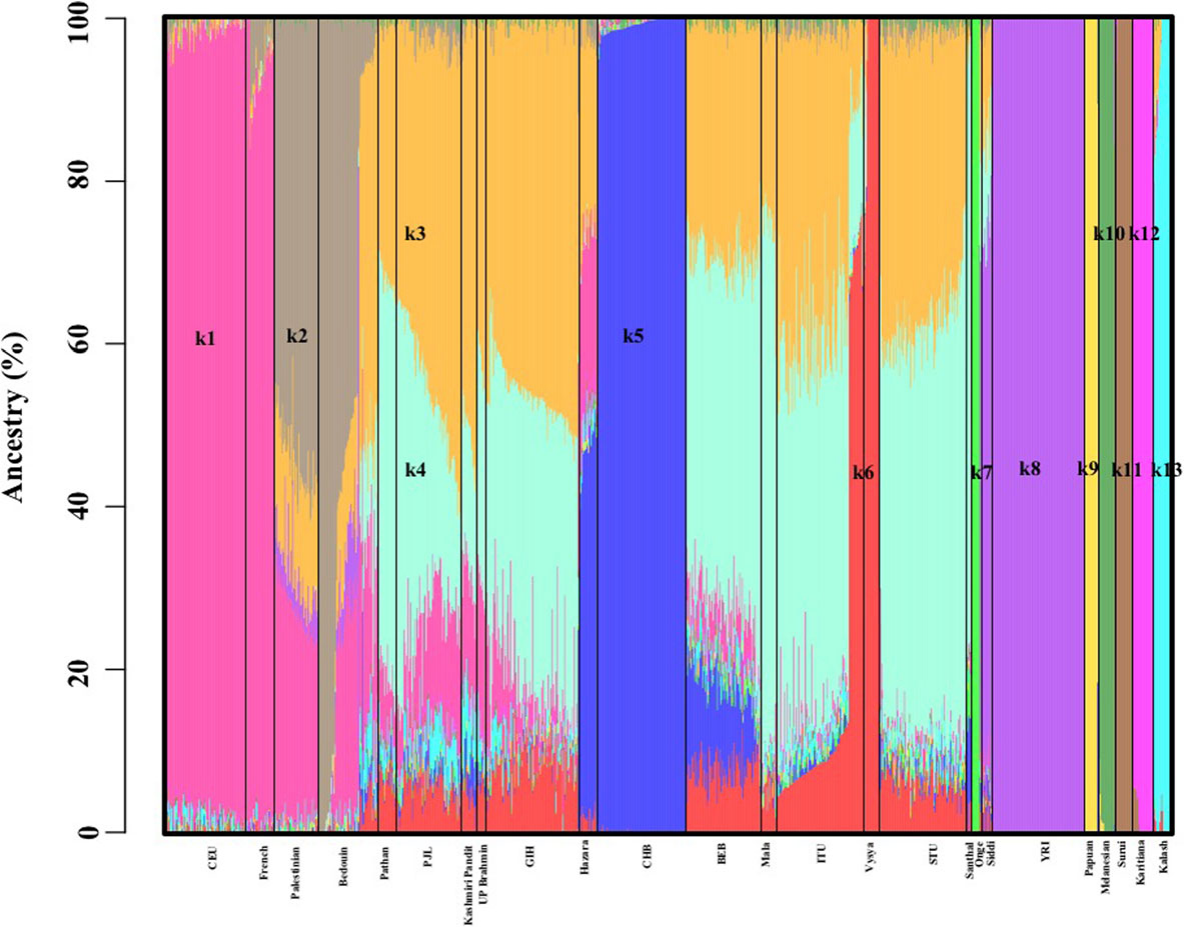
*An admixture plot showing the ancestry components of global populations*. Five SAS populations (STU, ITU, GIH, PJL and BEB) from 1000 Genomes Project [33], selected Indian [31] and Pakistani populations [34, 35] alongside French, Karitiana and Surui samples from Brazil, Melanesians, Papuans, Bedouins from Israel, Palestinians [34, 35], Northern and Western Europeans from Utah, USA (CEU), Han Chinese samples from Beijing, China (CHB), and Yorubans from Ibadan, Nigeria (YRI) [33], were evaluated. Percent ancestry is plotted on the Y axis. The ancestral components in evaluated genomes was estimated using ADMIXTURE v1.3. A model with thirteen ancestral components (*K* = 13) was the most parsimonious to explain the variation and similarities of the genome-wide genotype data. Each individual is represented by a vertical line partitioned into colored segments whose lengths are proportional to the contributions of the ancestral components to the genome of the individual. Population labels were added after each individual’s ancestry had been estimated. To note *k1, k2, k3, k4, k5, k6, k7, k8, k9, k10, k11, k12* and *k13* represent putative ancestral West Eurasian, Middle Eastern, North Indian, South Indian, Chinese, Telugu, Andamanese, African, Papuan, Melanesian, Surui, Karitiana, and Kalash components respectively

In the global admixture analysis, at *K* = 13, European (CEU and French), Chinese (CHB), African (YRI), Papuan, Melanesian, Surui and Karitiana samples were assigned homogeneously to unique populations putatively representing *k1*, *k5*, *k8*, *k9*, *k10*, *k11* and *k12* ancestral components (Fig. 1). While the ancestral components *k6* and *k7* predominantly occurred among the Telugu (Vysya) and Andamanese (Onge) populations. Finally, the ancestral component *k13* was exclusively found in the Kalash population that is a genomic isolate [41] from Pakistan. Most South Asians have high fractions of putative North Indian (*k3*) and South Indian (*k4*) ancestral components with variable fractions of *k1*, *k5* and *k6* in their genomes.

**Fig. 2:**
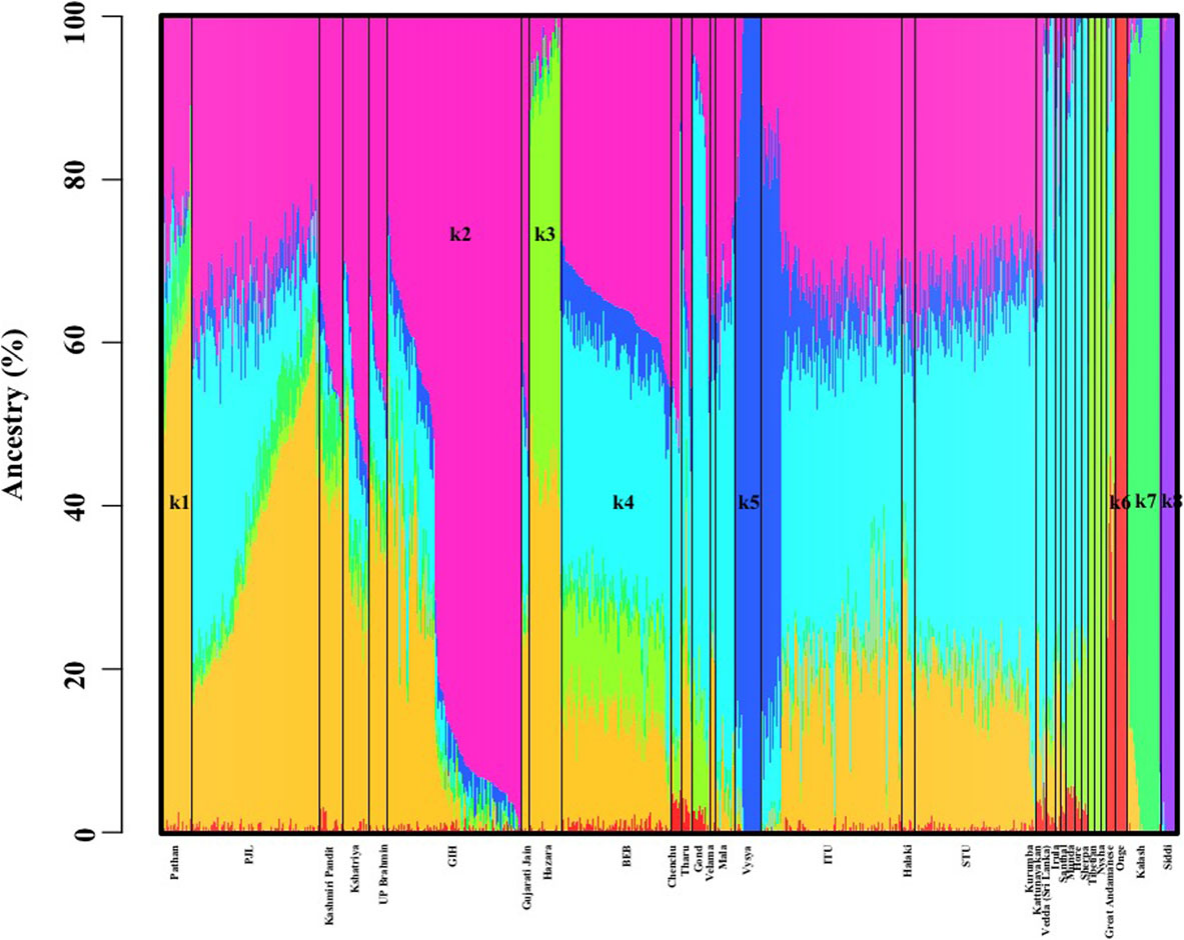
*An admixture plot showing the ancestry components of South Asian populations*. Five SAS populations (STU, ITU, GIH, PJL and BEB) from 1000 Genomes Project [33], and previously published Indian [31] and Pakistani populations [34, 35] were evaluated. Percent ancestry is plotted on the Y axis. The ancestral components in evaluated genomes was estimated using ADMIXTURE v1.3. A model with eight ancestral components (*K* = 8) was the most parsimonious to explain the variation and similarities of the genome-wide genotype data. Each individual is represented by a vertical line partitioned into colored segments whose lengths are proportional to the contributions of the ancestral components to the genome of the individual. Population labels were added after each individual’s ancestry had been estimated. To note *k1, k2, k3, k4, k5, k6, k7,* and *k8* represent putative ancestral West Eurasian, North Indian, East Asian, South Indian, Telugu, Andamanese, Kalash, and Siddi components respectively

**Fig. 3:**
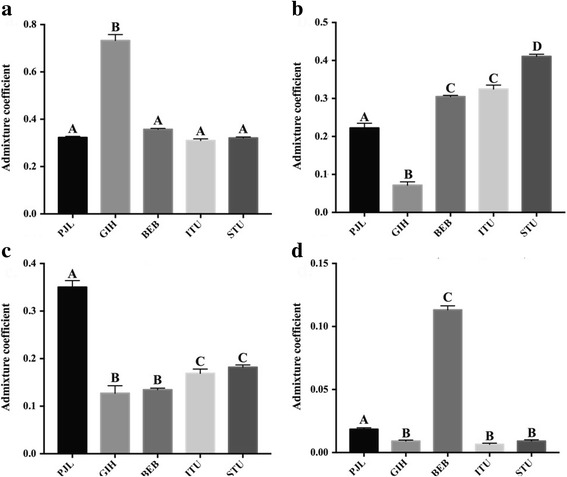
*Comparison of major admixture components among five SAS populations from 1000 Genomes Project*. Multiple comparisons were performed using Tukey’s post hoc analysis implemented in GraphPad Prism v7. **a** Comparison of North Indian component (*k2*). GIH had the highest North Indian ancestral component compared to the other four SAS populations (Tukey’s post hoc analysis, *p-value* < 0.0001). **b** Comparison of South Indian component (*k4*). STU had the highest fraction of South Indian component in their genome compared to the rest (Tukey’s post hoc analysis, *p-value* < 0.0001). **c** Comparison of West Eurasian component (*k1*). PJL possessed the highest fraction of West Eurasian component in their genome compared to the other four SAS populations (Tukey’s post hoc analysis, *p-value* < 0.0001). **d** Comparison of East Asian component (*k3*). BEB had the highest fraction of East Asian component in their genome compared to the rest (Tukey’s post hoc analysis, *p-value* < 0.0001)

In the South Asian only admixture analysis, at *K* = 8, a discernible degree of genetic admixture between the North and South Indian populations was evident from the admixture analysis (Fig. 2). Consistent with the global admixture studies and previous findings [31] the Punjabi (PJL), Gujarati (GIH), and ANI [31] genomes revealed a higher fraction of the North Indian ancestral component, *k2* (identical to component *k3* in the global analysis, Fig. 1), while the Tamil (STU), Telugu (ITU) and the ASI [31] genomes were demonstrated to possess a higher fraction of the South Indian ancestral component, *k4* as also observed in the global analysis. The GIH samples had the highest North Indian ancestral component, *k2* (Tukey’s post hoc analysis, *p-value* < 0.0001) (Fig. 3a), while the STU genomes possessed the highest levels of the South Indian component, *k4* (Tukey’s post hoc analysis, *p-value* < 0.0001) (Fig. 3b). The West Eurasian ancestry component, *k1* as also delineated in the global assessment was discerned to be the highest among Pathans, Kashmiri Pandits, Brahmins, Kshatriyas and PJL, while highest levels of the ancestral component, *k5* (same as the component *k6* in the global analysis, Fig. 1) was found in the genomes of the Telugu people including the highly endogamous Vysya group from Andhra Pradesh [32, 42] and the ITU population. In tune with their geographic origin and proximity to the West Eurasians, the highest fraction of the component *k1* was present in PJL when compared to the remaining four SAS populations (Tukey’s post hoc analysis, *p-value* < 0.0001) (Fig. 3c). As reported previously [43], the Bangladeshi (BEB) genomes were delineated to contain a significant fraction of the East Asian ancestral component, *k3* (identical to component *k5* in the global assessment, Fig. 1) (Tukey’s post hoc analysis, *p-value* < 0.0001) (Fig. 3d), in addition to discernable ‘North Indian’ and ‘South Indian’ ancestry components, which are likely linked to their geographical origin and migration history. The ancestral component *k6,* potentially representative of Andamanese ancestry (same as component *k7* in the global analysis, Fig. 1), was observed among most tribes from the Indian subcontinent in discernible proportions together with the South Indian component, *k4*. Finally the ancestral component *k8* as also found in the global studies in Fig. 1 was exclusively observed among the Siddis. Given that the Siddis are a unique Indian population of African ancestry [11, 44, 45] their genomic proximity to Yorubans is not unexpected.

### Biogeographical mapping of reference south Asian populations

**Fig. 4:**
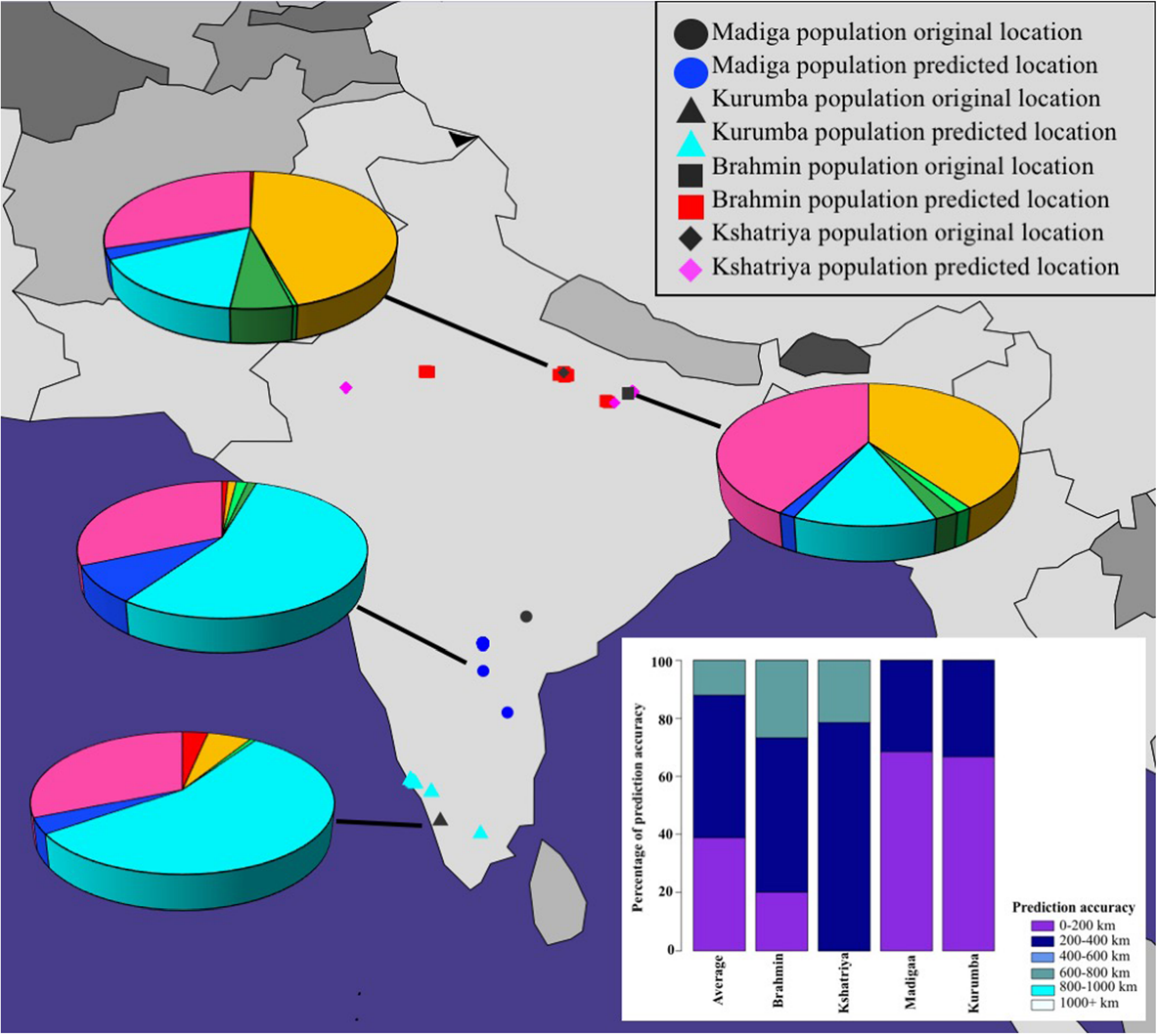
*GPS prediction of biogeographical affinities for four Indian reference populations: Brahmin, Kshatriya, Madiga, and Kurumba*. The original locations corresponding to all four populations are shown in black - square, diamond, circle and triangle respectively. The GPS predicted coordinates of Brahmins, Kshatriyas, Madigas, and Kurumbas are shown in red squares, purple diamonds, blue circles and cyan triangles, respectively. Pie charts reflect the admixture proportions of each of the four reference populations. The colors in the pie charts correspond to those used to represent the various admixture components depicted in Fig. 2. Inset shows stacked bar plots depicting the accuracy of GPS predictions for the four reference populations. *Note*: in some cases, multiple individuals from certain populations were assigned to the same location and therefore appeared as a single individual. Maps were plotted using the R package rworldmap v1.3–1 [52]

Prior to applying GPS to elucidate the biogeographical affinity of South Asian populations, we sought to trial its accuracy on 57 individuals belonging to four Indian populations (Brahmin, Kshatriya, Madiga, and Kurumba) corresponding to known latitudes and longitudes, as described previously [31]. We applied GPS using the *leave-one-out* procedure [9, 10] at the population level. Assignment accuracy was determined for each of the 57 individuals. GPS correctly assigned 88% of all individuals to less than or equal to 600 km from their original location (Fig. 4). For both South Indian populations, Madiga and Kurumba, 100% of all individuals were correctly assigned within 400 km of their known regional location while 73% Brahmin and 78% Kshatriya groups were assigned within 600 km of their original geographic region. These results demonstrate a reasonably strong geographic-genomic correspondence and delineate the expected assignment error for the SAS populations.

### Biogeographical mapping of south Asian populations from 1000 genomes project

**Fig. 5:**
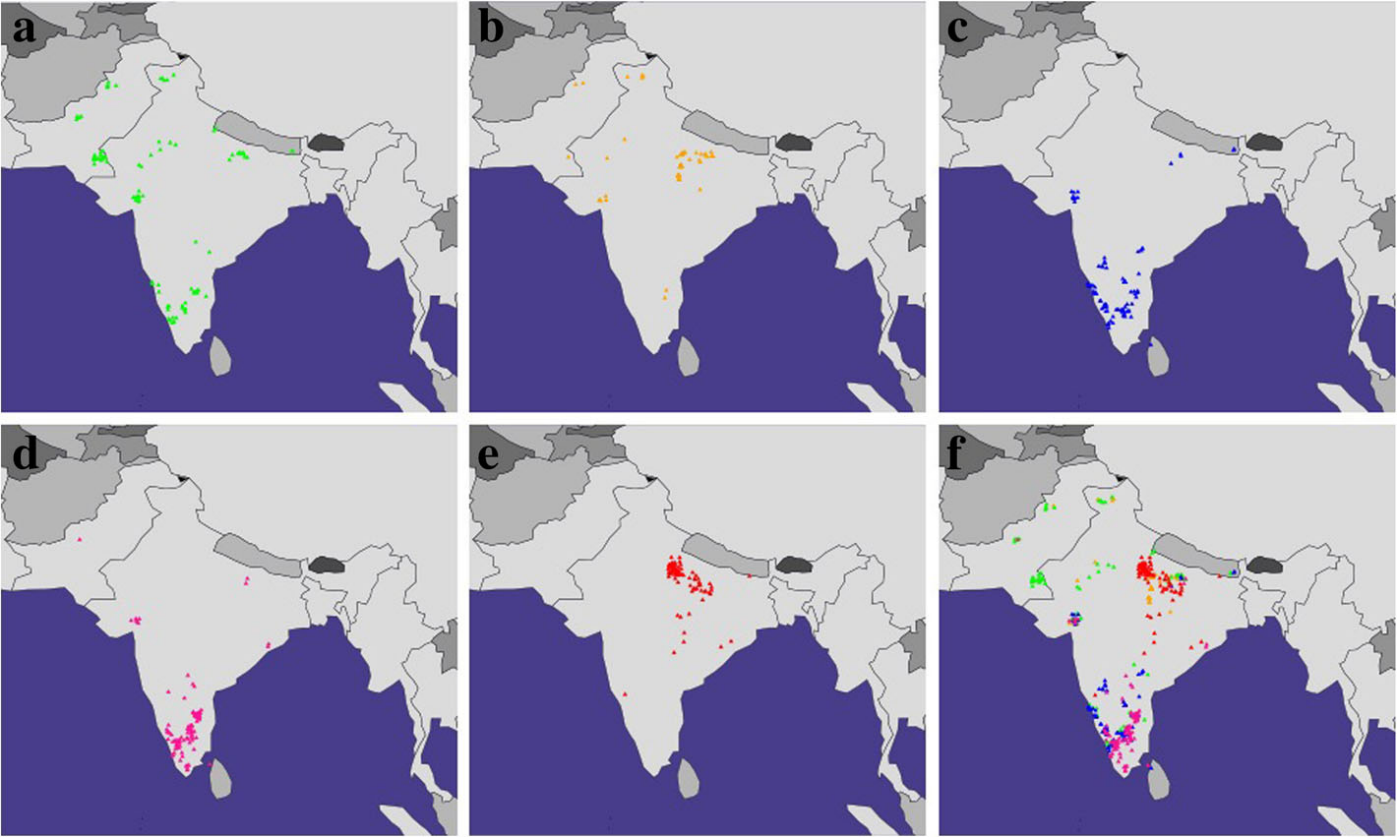
*GPS predictions for the five SAS populations from 1000 Genomes Project*. A map depicting the GPS predicted locations for **(a)** PJL **(b)** GIH **(c)** ITU **(d)** STU **(e)** BEB and **(f)** merged. The red, blue, orange, green, and pink triangles depict BEB, ITU, GIH, PJL and STU populations, respectively. Maps were plotted using the R package rworldmap v1.3–1 [52]

Next, we applied the GPS algorithm to infer the biogeographical affinity of the five SAS populations from 1000 Genomes Project [33] using South Asian populations described earlier [31, 34, 35] as the reference. GPS assigned locations for the query SAS populations are depicted in Fig. 5. As evident most SAS genomes were positioned in continental India, Pakistan and the remaining in Sri Lanka (Additional file 1: Table S1). A majority of Punjabi, PJL (>70%) and Gujarati, GIH (>90%) genomes were positioned in northern and western regions of the Indian subcontinent, across western Pakistan and the Indian states of Jammu and Kashmir, Uttarakhand, Uttar Pradesh, Rajasthan and Gujarat (Fig. 5a, b). We note that the remainder of the PJL individuals was assigned across the southern Indian states of Karnataka, Kerala and Andhra Pradesh (Fig. 5a), while the remaining GIH individuals were positioned in Andhra Pradesh (Fig. 5b). As may be surmised most genomes corresponding to that of the south Indian groups, STU and ITU (>80%) were positioned across the southern Indian states of Andhra Pradesh, Telangana, Tamil Nadu, Kerala, and Karnataka with those remaining being positioned in northern India (Fig. 5c, d). Except a single Bangladeshi, BEB individual who was positioned in Karnataka, the remainder of the BEB genomes was localized in northern and central India, spread across the states of Uttarakhand, Uttar Pradesh, Madhya Pradesh, Chhattisgarh, and Orissa (Fig. 5e).

**Fig. 6:**
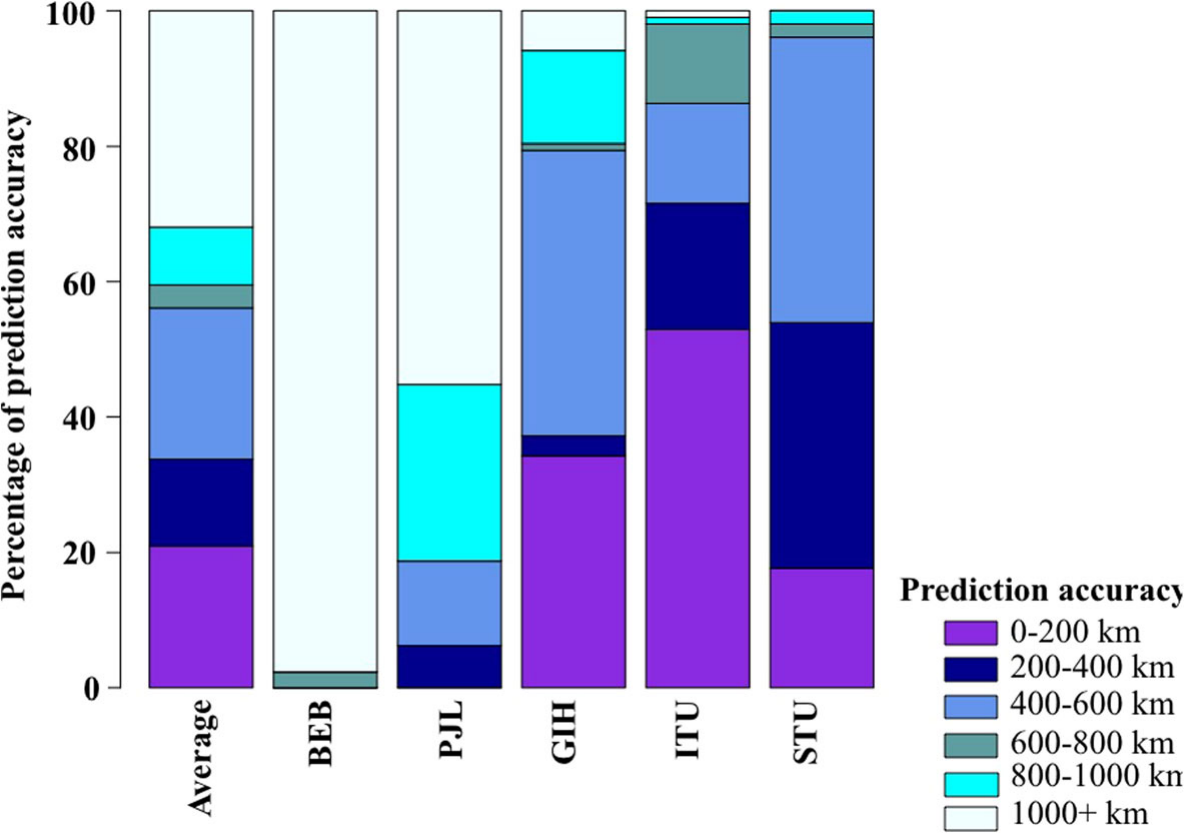
*Stacked bar plots representing the assignment accuracy of GPS algorithm*. Blue-violet, dark-blue, cornflower-blue, cadet-blue, cyan, and azure segments represent the positioning of STU, ITU, GIH, PJL and BEB populations within 200 km, 400 km, 600 km, 800 km, 1000 km and more than 1000 km, respectively from their corresponding native locations. The geographical coordinates (latitude and longitude) of Ahmedabad (India), Hyderabad, (India), Colombo (Sri Lanka), Lahore (Pakistan), and Dhaka (Bangladesh) were used as the native locations for GIH, ITU, STU, PJL and BEB respectively for the geographic distance estimation

GPS assignment accuracy was ascertained for each SAS individual to investigate the proximity of their predicted location to that of their likely regional location. Overall more than 56% and 88% of all queried genomes were positioned within 600 km and 1000 km, respectively from their regional location (Fig. 6). The GPS predictions were most accurate for STU and ITU individuals, where 96% and 87% of samples, respectively, were positioned within 600 km of their native locations, Hyderabad (India) and Colombo (Sri Lanka), respectively. Likewise, a substantial number of GIH individuals (~79%) were positioned within 600 km of their regional location, Ahmedabad (India). In contrast, the GPS assignments were only moderate-low in accuracy for PJL and BEB populations. ~ 19% PJL genomes were positioned within 600 km of their native location, Lahore (Pakistan), while a majority were assigned largely across northern and western India, the remainder were positioned in southern India. This is a likely consequence of the high genomic similarity of PJL individuals with Indian populations compared to its geographically proximal Pakistani populations. We also observed poor assignment accuracy for the Bengali individuals, wherein ~ 98% BEB genomes were assigned to locations more than 1000 km of their native region.

## Discussion

The utilization of biological data to infer the geographic origins of human populations has piqued the curiosity of geneticists and anthropologists for decades. Presently several biogeographical approaches using high-resolution next-generation sequencing data are available that are based on identity by distance, nevertheless the accurate geo-localization of populations has remained a challenge. GPS has been used successfully for determination of the biogeographical affinity of several worldwide populations [2, 6, 7, 9–11]. This approach correlates the admixture proportions of the query populations with that of the reference groups known to have resided in a specific geographic location for a substantial period of time and infers the geographic coordinates (latitude and longitude) of the former based on the geographic information pertaining to the latter. The admixture signature of a population maybe modulated by genetic exchanges with other groups during its migration while its isolation and segregation preserves its original admixture signal. Therefore, unmixed populations are likely to be localized more reliably close to their best matching reference by the GPS algorithm, however, for populations where the admixture event is recent GPS predictions are likely to be error-prone [2, 6].

India has served as a prominent corridor for the migration of anatomically modern humans in the Paleolithic and Neolithic era [13–15, 19, 46]. Its demographic history has been modulated by multiple, large-scale population migrations, admixture and the regimented sociocultural enforcement of practices like endogamy [21–24, 26, 30, 32, 47–50]. Given the complex population history and genetic heterogeneity of the Indian subcontinent, here we sought to examine the effectiveness of the GPS approach for localizing five SAS populations obtained from the 1000 Genomes project [33] using populations from the Indian subcontinent available in HGDP [34, 35] and those previously described [31] as reference.

Our trial analysis of four Indian groups, Brahmin, Kshatriya, Madiga, and Kurumba [31] using the *leave-one out* procedure demonstrated strong genomic-geographic relationship with 88% of all the individuals being assigned within 600 km of their regional location (Fig. 4). Among our query dataset GPS appeared to position three SAS populations, Tamil (STU), Telegu (ITU) and Gujarati (GIH) with reasonably high accuracy. Notably all three populations corresponded to the South Asian diaspora and were sampled from UK and USA respectively. Consistent with expectation STU and ITU populations from the southern region of the Indian subcontinent possessed very high South Indian ancestral component, *k4* (Fig. 3b). Overall they were largely (>80%) positioned across the southern Indian states of Andhra Pradesh, Telangana, Tamil Nadu, Kerala, and Karnataka (Fig. 5c, d). We obtained high accuracy for the GPS assignment of STU and ITU individuals, where 96% and 87% of individuals respectively, were positioned within 600 km of their native locations, Hyderabad (India) and Colombo (Sri Lanka), respectively. Similar results were derived for the GIH population, which contained the highest North Indian ancestral component, *k2* (Fig. 3a); >90% of the GIH individuals were assigned across northern and western regions of the Indian subcontinent (Fig. 5b). GPS placed ~79% of the GIH individuals within 600 km of their regional location, Ahmedabad (India). We note that with the presently utilized GPS algorithm only a handful of Gujarati samples were positioned within the state of Gujarat, which is in concordance with a previous study where approximately 25% GIH individuals were mapped to their native location using GPS [51], thereby underscoring the genetic heterogeneity in the GIH genomes sampled in the 1000 Genomes project. In contrast, GPS predictions were moderately accurate for the Punjabi (PJL) population, ~ 19% PJL genomes were positioned within 600 km of their native location, Lahore (Pakistan), while a majority were assigned across western and northern regions of the Indian subcontinent and the remainder were positioned in southern India (Fig. 5a). While the PJL population contained the highest fraction of the ancestral West Eurasian component, *k1* (Fig. 3c), it also possessed substantial North and South Indian ancestral components, *k2* and *k4* respectively (Figs. 2 and 3a, b). We note that the PJL genomes possessed a significantly higher fraction of the *k4* ancestral component compared to the other North Indian population, GIH (Tukey’s post hoc analysis, *p-value* < 0.0001) (Fig. 3b) which likely accounts for the higher number of PJL individuals being assigned to the southern regions of the Indian subcontinent compared to GIH (Fig. 5a, b). Further PJL genomes contained the highest West Eurasian ancestral component, *k1* among the query SAS populations (Fig. 3c), while it’s North Indian ancestral component, *k2* appeared substantially lower than that of the GIH group (Fig. 3a). We surmise that the high proportions of *k1* component present among Pathans, Kashmiri Pandits, Brahmins and Kshatriyas (Fig. 2) in our reference dataset likely led to a greater proportion of PJL individuals being localized towards the north-western regions of the Indian subcontinent (Fig. 5a). The presence of high West Eurasian component (*k1*) in Brahmins and Kashmiri Pandits is in concordance with a previous study that demonstrated the presence of 11.4% and 10.6% of Northern Eurasian and Mediterranean components, respectively, in the Brahmin genome [51]. As speculated in Arunkumar et al. (2015) [51] a higher fraction of *k1* in Brahmins and North-West Indians indicates a potential shared ancestry between the Brahmins and Europeans. Finally the GPS assignment accuracy was found to be poor for the Bengali (BEB) individuals, wherein ~ 98% BEB genomes were assigned to locations more than 1000 km of their native region, Dhaka (Bangladesh) (Fig. 5e). We note that BEB genomes contained the highest East Asian and discernible North and South Indian ancestral components, *k3, k2* and *k4* respectively (Fig. 2) which in the absence of appropriate reference populations in our analysis lead the GPS tool to interpret them in accordance with their genetic similarity with the available reference groups and localized the BEB individuals at average geographic locations where a consensus appeared to be reached. The presence of high East Asian component (*k3*) among Bengalis and Northeast Indians supports the presence of at least three [51] to four [22] major admixture components in Indian genome, contrary to a more popular two component theory [32].

Inference of the geographic origin of individuals on the basis of their genetic information poses a formidable challenge and is prone to misinterpretations. In this study the GPS algorithm employed the unsupervised ADMIXTURE analysis where an appropriate number of admixture components for the model in question is deduced, evaluating both the query and reference populations against the same putative ancestral populations. GPS correlates the relative proportions of admixture in the query and reference populations to extrapolate the geographic location of the former on the basis of the geographic coordinates (latitude and longitude) of the latter. Therefore, the reference populations can be conceived as drawing the query groups on the basis of the corresponding genetic proximity till an agreement of geographical locations is achieved [6]. Our results shed light on the efficacy and limitations of the GPS tool for biogeographical analyses of admixed populations, using the evaluation of SAS groups from 1000 Genomes project as a case in point. Here we note that although the 1000 Genomes project is an invaluable resource of publically available WGS data for a wide range of ethno-linguistic groups, the use of language as a proxy for an ethnic unit renders the SAS diaspora samples as likely suboptimal representatives of populations from the Indian subcontinent [43]. We surmise that the lack of precision and adequate corrective measures in sampling individuals with well-defined SAS ancestries in the 1000 Genomes project together with our assumption of the capital cities of their putative country or ancestral region as their likely origin, may have further constrained the assignment accuracy of the GPS model. Since GPS predictions are likely the last location where admixture had occurred or the geographic origin, for individuals of mixed ancestries, GPS assignments represent the mean geographic locations of their immediate parental populations. However, given the present GPS model we observed reasonably high accuracy in its assignments even for recent migrant populations sampled elsewhere, STU, ITU and GIH. However, its predictive capacity was severely curtailed when suitable reference groups were unavailable, as in case of the BEB individuals. Nevertheless, we envision that as WGS data becomes available from a greater number of populations worldwide and a better reference panel will be available to map the ancestry of currently unresolved populations such as the Bengali individuals from Bangladesh.

While in theory the GPS approach can have putative applications in various fields of science, including genealogical research, where GPS can aid adopted individuals to localize their home regions; it may be employed in forensics, where it can improve the assignment of geographic ancestry to DNA evidence [2]. Further we surmise that due to its inherent ability to detect correlation between genomic and geographic information, the GPS approach can potentially be used to investigate local adaptations. However, its administration and efficacy in the realms of these applications is yet to be evaluated.

## Conclusions

Despite its success in tracing ancestry of several modern-day populations and several other likely applications, our findings exemplify that the GPS approach is heavily dependent on the relative proportions of admixture in the reference populations to articulate the population history and biogeographical origins of test individuals. Given the perils of bias in the GPS predictions, interpretation of its results must be performed with adequate caution. Finally, we conclude that further modifications are desired to make this approach more suitable for highly admixed individuals.

## Additional file

Additional file 1:
**Table S1.** GPS predicted coordinates of individuals from five SAS populations. **Figure S1.** (a) Table showing proportion of Cross-Validation error (CVE) in ADMIXTURE carried out for the global dataset with different values of ancestral components (K) employed in the admixture analysis. The CVE was used to determine the optimum number of ancestral components (K) supported by the data. At *K* = 13 the CVE was minimized. (b) Plot depicting the change of CVE with increasing number of ancestral components (K). The optimum number of ancestral components with lowest CVE was thirteen (*K* = 13). **Figure S2.** (a) Table showing proportion of Cross-Validation error (CVE) in ADMIXTURE carried out for the South Asian only dataset with different values of ancestral components (K) employed in the admixture analysis. The CVE was used to determine the optimum number of ancestral components (K) supported by the data. At *K* = 8 the CVE was minimized. (b) Plot depicting the change of CVE with increasing number of ancestral components (K). The optimum number of ancestral components with lowest CVE was 8 (*K* = 8). (PDF 493 kb)
