# Correction to: Application of geographic population structure (GPS) algorithm for biogeographical analyses of populations with complex ancestries: a case study of South Asians from 1000 genomes project

**DOI:** 10.1186/s12863-018-0683-y

**Published:** 2018-10-25

**Authors:** Ranajit Das, Priyanka Upadhyai

**Affiliations:** 1 Manipal Centre for Natural Sciences (MCNS), Manipal Academy of Higher Education, Madhav Nagar, Manipal, Karnataka 576104 India; 2 Department of Medical Genetics, Kasturba Medical College, Manipal Academy of Higher Education, Manipal, Karnataka India

## Correction

Following publication of the original article [1], the authors flagged that acknowledgment of their equal contribution is omitted in the article [1].

As such, please be advised that both authors contributed equally to this work.

We apologize for this processing error.
