# Supplementary material for: Application of geographic population structure (GPS) algorithm for biogeographical analyses of populations with complex ancestries: a case study of South Asians from 1000 genomes project
Source: BMC Genet. 2017 Dec 28;18(Suppl 1):109. doi: 10.1186/s12863-017-0579-2 (PMC5751663; doi:10.1186/s12863-017-0579-2)
Supplement: Additional file 1: — Table S1. GPS predicted coordinates of individuals from five SAS populations. Figure S1. (a) Table showing proportion of Cross-Validation error (CVE) in ADMIXTURE carried out for the global dataset with different values of ancestral components (K) employed in the admixture analysis. The CVE was used to determine the optimum number of ancestral components (K) supported by the data. At K = 13 the CVE was minimized. (b) Plot depicting the change of CVE with increasing number of ancestral components (K). The optimum number of ancestral components with lowest CVE was thirteen (K = 13). Figure S2. (a) Table showing proportion of Cross-Validation error (CVE) in ADMIXTURE carried out for the South Asian only dataset with different values of ancestral components (K) employed in the admixture analysis. The CVE was used to determine the optimum number of ancestral components (K) supported by the data. At K = 8 the CVE was minimized. (b) Plot depicting the change of CVE with increasing number of ancestral components (K). The optimum number of ancestral components with lowest CVE was 8 (K = 8). (PDF 493 kb) [file 12863_2017_579_MOESM1_ESM.pdf]

Supplementary data

Table S1: GPS predicted coordinates of individuals from five SAS populations

Sample id	Population	Genetically closest reference individual (from Moorjani et al. 2013 and HGDP)	GPS predicted Latitude	GPS predicted Longitude
NA20845	GIH	Vaish_132_R1	25.7053214	81.8308755
NA20846	GIH	KSHA_UP3M	25.91815	80.8574006
NA20847	GIH	KSHA_UP4S	25.2954855	80.0223729
NA20849	GIH	KSHA_UP4S	25.3763908	80.0461121
NA20850	GIH	Kashmiri_Pandit_123_R1	33.5182411	76.5875847
NA20851	GIH	KSHA_UP4S	25.339942	80.04426
NA20852	GIH	KSHA_UP3M	26.3957595	80.0302172
NA20853	GIH	KSHA_UP4S	25.3152389	80.0326651
NA20854	GIH	KSHA_UP5F	26.5525596	81.4564836
NA20856	GIH	KSHA_UP4S	26.4232219	80.1624404
NA20858	GIH	PAND_JK5D	33.4735619	75.1227662
NA20859	GIH	KSHA_UP4S	25.2461313	80.0108907
NA20861	GIH	KSHA_UP4S	24.4346099	79.7965519
NA20862	GIH	PAND_JK5M	33.3752459	76.4537832
NA20863	GIH	KSHA_UP4S	25.3570876	80.0488964
NA20864	GIH	BRAH_UP3M	26.4275031	81.9238292
NA20866	GIH	KSHA_UP4M	26.3887982	80.0989162
NA20867	GIH	KSHA_UP4S	25.3822221	80.0403137
NA20868	GIH	KSHA_UP4S	25.2875528	80.021263
NA20869	GIH	KSHA_UP4S	25.2859732	80.0198835
NA20870	GIH	HGDP00230Pathan	32.7905961	70.3669868
NA20872	GIH	KSHA_RJ02	27.7422634	74.8644206
NA20874	GIH	Meghawal_119_R2	26.0639216	73.2454748
NA20875	GIH	KSHA_UP4S	24.115395	79.9033297
NA20876	GIH	KSHA_UP4S	26.4063344	80.1312768
NA20877	GIH	KSHA_UP4S	26.4025809	80.1243503
NA20878	GIH	KSHA_UP4S	25.2825091	80.0168785
NA20881	GIH	BRAH_UP3M	26.4545783	82.2933113
NA20882	GIH	BRAH_UP2F	26.3150399	82.2961161
NA20884	GIH	KSHA_UP4S	24.4630029	79.7971512
NA20885	GIH	Vaish_133_R1	26.1827798	82.4661992
NA20886	GIH	BRAH_UP2S	26.6178217	82.8317037
NA20887	GIH	KSHA_UP1M	26.6248189	80.3713858
NA20888	GIH	KSHA_UP4S	24.5058295	79.8145983
NA20889	GIH	KSHA_UP4S	26.4259183	80.1674162
NA20890	GIH	BRAH_UP3M	26.2297886	82.8581834
NA20891	GIH	BRAH_UP2F	26.2235489	83.0084329
NA20892	GIH	KSHA_UP4S	25.3780806	80.0647997

Sample id	Population	Genetically closest reference individual (from Moorjani et al. 2013 and HGDP)	GPS predicted Latitude	GPS predicted Longitude
NA20894	GIH	Vaish_132_R1	25.8700261	81.7406937
NA20895	GIH	KSHA_UP3M	26.5817171	80.4221885
NA20896	GIH	KSHA_UP4S	25.3085947	80.0341705
NA20897	GIH	KALL_TN01	13.217453	78.5656215
NA20899	GIH	KSHA_UP4S	25.2562669	80.0094379
NA20900	GIH	BRAH_UP2F	26.2366352	82.9183761
NA20901	GIH	JAIN_GU01	22.1993046	72.5649649
NA20902	GIH	KSHA_UP4S	25.2644283	80.0079237
NA20903	GIH	KSHA_UP3F	26.1634928	79.6831465
NA20904	GIH	HGDP00201Sindhi	26.1640895	69.6878258
NA20905	GIH	JAIN_GU01	22.1881528	72.5706879
NA20906	GIH	Naidu_177_R2	13.9251453	78.7036711
NA20908	GIH	BHIL_MP03	22.1870912	73.1570531
NA20910	GIH	HGDP00239Pathan	32.9084052	71.0451086
NA20911	GIH	KSHA_UP4S	25.447549	80.0544688
NA21086	GIH	KSHA_UP4S	24.4269093	79.9287296
NA21087	GIH	KSHA_UP4S	24.067551	79.8630805
NA21088	GIH	KSHA_UP4S	26.4181748	80.1531266
NA21089	GIH	KSHA_UP4S	25.3539049	80.0348765
NA21090	GIH	KSHA_UP4S	24.4074828	79.7829577
NA21091	GIH	KSHA_UP4S	25.3328417	80.0134216
NA21092	GIH	Srivastava_198_R2	23.178739	81.8034143
NA21093	GIH	KSHA_UP4S	25.3061326	80.0315697
NA21094	GIH	KSHA_UP4S	25.3512167	80.0327709
NA21095	GIH	JAIN_GU01	22.1360036	72.6363441
NA21097	GIH	JAIN_GU05	22.2097943	72.5725717
NA21098	GIH	KSHA_UP4S	24.5178412	79.8190677
NA21099	GIH	KSHA_UP4S	26.4287413	80.1726257
NA21100	GIH	KSHA_UP4S	25.2952799	80.0259309
NA21101	GIH	KSHA_UP4S	24.4589957	79.8674074
NA21102	GIH	KSHA_UP4S	24.0969129	79.8738012
NA21103	GIH	KSHA_UP4S	25.3172842	80.0341701
NA21104	GIH	KSHA_UP4S	25.2893437	80.0200174
NA21105	GIH	KSHA_UP4S	26.419553	80.1556699
NA21106	GIH	KSHA_UP4S	25.3651778	80.0392815
NA21107	GIH	KSHA_UP4S	26.4317314	80.1781435
NA21108	GIH	Kashmiri_Pandit_125_R1	33.6423778	76.4789876
NA21109	GIH	KSHA_UP4S	25.3021602	80.0245631
NA21110	GIH	KSHA_UP4S	25.2966235	80.0255497
NA21111	GIH	KSHA_UP4S	26.4306112	80.1760764
NA21112	GIH	KSHA_UP4S	25.2815013	80.0235469

Sample id	Population	Genetically closest reference individual (from Moorjani et al. 2013 and HGDP)	GPS predicted Latitude	GPS predicted Longitude
NA21113	GIH	KSHA_UP4S	24.0963733	79.8950045
NA21114	GIH	KSHA_UP4S	26.3964101	80.1129631
NA21115	GIH	KSHA_UP4S	25.3317089	80.0419788
NA21116	GIH	JAIN_GU03	22.5735321	73.1212399
NA21117	GIH	KSHA_UP4S	26.411494	80.1407982
NA21118	GIH	KSHA_UP4S	25.2665857	80.0086407
NA21119	GIH	KSHA_UP4S	25.2793209	80.0142167
NA21120	GIH	KSHA_UP1F	26.576936	80.3674752
NA21122	GIH	KSHA_UP4S	25.2977678	80.0265962
NA21123	GIH	KSHA_UP4S	26.4046565	80.1281806
NA21124	GIH	KSHA_UP4S	26.4199456	80.1563945
NA21125	GIH	KSHA_UP4S	25.3095468	80.0321118
NA21126	GIH	KSHA_UP4S	25.3239699	80.0276099
NA21127	GIH	KSHA_UP4S	25.346621	80.0440759
NA21128	GIH	KSHA_UP4S	25.3440262	80.0402684
NA21129	GIH	KSHA_UP4S	25.2867412	80.0255767
NA21130	GIH	KSHA_UP4S	26.4097974	80.1376674
NA21133	GIH	KSHA_UP4S	25.2737974	80.0146392
NA21135	GIH	KSHA_UP4S	25.2613188	80.0063054
NA21137	GIH	KSHA_UP4S	25.3064402	80.0203196
NA21141	GIH	KSHA_UP4S	25.2879037	80.0223609
NA21142	GIH	KSHA_UP4S	25.3659633	80.0611091
NA21143	GIH	KSHA_UP4S	25.3113031	80.0350522
NA21144	GIH	KSHA_UP4S	25.3041983	80.0285709
HG01583	PJL	BRAH_UP2F	26.1823444	82.9854111
HG01586	PJL	BRAH_UP3M	26.4588065	82.5138339
HG01589	PJL	PAND_JK5D	33.6969452	76.4296392
HG01593	PJL	HGDP00201Sindhi	25.8714173	69.7391366
HG02490	PJL	HGDP00169Sindhi	26.5953092	69.7013976
HG02491	PJL	HGDP00070Balochi	29.7527623	67.7140349
HG02493	PJL	HGDP00169Sindhi	26.1248397	69.1925535
HG02494	PJL	PAND_JK5M	33.3717715	75.2203745
HG02597	PJL	KSHA_RJ02	26.8718969	75.1988938
HG02600	PJL	JEWS_KE05	11.002077	76.4702725
HG02601	PJL	Meghawal_113_R1	25.4577438	73.2356033
HG02603	PJL	Tharu_104_R1	28.8128057	80.3361191
HG02604	PJL	HGDP00167Sindhi	25.9165391	69.0781011
HG02648	PJL	HGDP00258Pathan	32.7202735	71.1337218
HG02649	PJL	HGDP00230Pathan	32.5518647	70.3222246
HG02651	PJL	KSHA_RJ01	27.290974	76.6012722
HG02652	PJL	BRAH_UP3S	26.5188305	82.3812464

Sample id	Population	Genetically closest reference individual (from Moorjani et al. 2013 and HGDP)	GPS predicted Latitude	GPS predicted Longitude
HG02654	PJL	HGDP00189Sindhi	25.9756789	69.290624
HG02655	PJL	HGDP00210Sindhi	26.2277684	69.1364566
HG02657	PJL	Meghawal_119_R2	26.3700134	74.3372076
HG02658	PJL	HGDP00070Balochi	29.8848781	67.9163964
HG02660	PJL	BRAH_UP2F	26.4555556	82.9096567
HG02661	PJL	Tharu_104_R1	28.5663721	80.1336187
HG02681	PJL	HGDP00203Sindhi	26.1648116	70.1266601
HG02682	PJL	HGDP00205Sindhi	25.7656023	70.2790352
HG02684	PJL	Hallaki_140_R2	13.7409733	75.0792289
HG02685	PJL	KVGO_TN04	12.4494192	77.228349
HG02687	PJL	Naidu_177_R2	13.9278888	78.7055157
HG02688	PJL	BHIL_MP05	22.4755767	73.0278908
HG02690	PJL	HGDP00183Sindhi	26.0263305	69.098687
HG02691	PJL	HGDP00210Sindhi	25.5394098	69.1165195
HG02694	PJL	HGDP00187Sindhi	26.3447026	69.1583817
HG02696	PJL	JAIN_GU02	22.4731792	72.7756616
HG02697	PJL	PAND_JK5M	33.3846573	75.4154846
HG02699	PJL	KSHA_RJ04	27.5493036	75.0895541
HG02700	PJL	HGDP00208Sindhi	25.9793009	69.7802418
HG02724	PJL	MADI_AP3M	18.364046	78.4696921
HG02725	PJL	BHIL_MP05	22.0644482	73.1959255
HG02727	PJL	BHIL_MP01	22.4599064	73.0665739
HG02728	PJL	Naidu_177_R2	14.0139625	78.0564611
HG02731	PJL	HGDP00070Balochi	29.6090412	67.4753508
HG02733	PJL	BRAH_UP2F	26.5784031	82.8100214
HG02734	PJL	HGDP00167Sindhi	26.0094315	69.0955184
HG02736	PJL	PAND_JK5M	33.2022005	75.5477528
HG02737	PJL	HGDP00203Sindhi	26.2137449	69.9736185
HG02774	PJL	JEWS_KE02	11.2820458	75.9701508
HG02775	PJL	BHIL_MP01	22.2243735	73.2537634
HG02778	PJL	BHIL_MP05	22.5461848	73.2465348
HG02780	PJL	BHIL_MP05	22.5435395	73.0844277
HG02783	PJL	KVGO_TN05	11.8630792	77.5127416
HG02784	PJL	BHIL_MP03	23.0593946	73.2207755
HG02786	PJL	MADI_AP3M	17.4299563	79.720636
HG02787	PJL	Naidu_178_R2	13.6649049	78.5728297
HG02789	PJL	BHIL_MP05	22.6070941	73.5845454
HG02790	PJL	Naidu_178_R2	13.6834783	78.5660602
HG02792	PJL	NARI_TN02	13.3502255	79.4193573
HG02793	PJL	Naidu_177_R2	13.764989	78.2930505
HG03015	PJL	KVGO_TN03	12.0564201	77.4499796

Sample id	Population	Genetically closest reference individual (from Moorjani et al. 2013 and HGDP)	GPS predicted Latitude	GPS predicted Longitude
HG03016	PJL	Naidu_178_R2	13.6444893	78.3001682
HG03018	PJL	KVGO_TN04	12.1732173	77.5138057
HG03019	PJL	Kurumba_44_R1	11.3593075	76.3439469
HG03021	PJL	Naidu_178_R2	13.7683191	78.5150933
HG03022	PJL	JEWS_KE05	11.0846632	76.0837222
HG03228	PJL	Hallaki_139_R2	14.4962984	74.4686971
HG03229	PJL	Hallaki_140_R2	14.3918662	75.0269646
HG03234	PJL	PAND_JK5M	33.2282032	75.8264382
HG03235	PJL	BRAH_UP2S	26.5892155	82.5110664
HG03237	PJL	HGDP00189Sindhi	26.0734696	69.5319943
HG03238	PJL	HGDP00189Sindhi	26.197422	69.1520856
HG03488	PJL	BRAH_UP2F	26.1391003	83.2183696
HG03490	PJL	HGDP00201Sindhi	26.3318365	69.7371227
HG03491	PJL	KVGO_TN02	12.7312614	77.5077434
HG03619	PJL	HGDP00228Pathan	32.7532708	70.3599883
HG03624	PJL	HGDP00201Sindhi	26.7419867	69.9135747
HG03625	PJL	HGDP00230Pathan	33.0623861	70.4179474
HG03629	PJL	KSHA_RJ01	27.4804729	76.1086468
HG03631	PJL	BRAH_UP3M	26.3807879	81.8460913
HG03634	PJL	HGDP00208Sindhi	26.0818871	69.9382845
HG03636	PJL	BRAH_UP3M	26.5292318	82.4258978
HG03640	PJL	KURU_KE02	12.443413	76.0091181
HG03649	PJL	Vaish_135_R1	25.9152046	81.585823
HG03652	PJL	JEWS_KE05	10.8917309	76.2993721
HG03653	PJL	JAIN_GU02	22.4344972	72.7069199
HG03660	PJL	Hallaki_145_R2	14.6706527	74.4283699
HG03663	PJL	KURU_KE02	12.1283483	75.9087494
HG03667	PJL	KURU_KE04	12.3879288	75.8777892
HG03668	PJL	Meghawal_119_R2	25.3049961	73.4513707
HG03702	PJL	SHER_WB01	26.7377164	87.3556791
HG03703	PJL	SHER_WB01	26.712657	87.370311
HG03705	PJL	Naidu_178_R2	13.8755877	78.6283673
HG03706	PJL	KVGO_TN04	12.2601923	77.2230456
HG03708	PJL	HGDP00070Balochi	29.8784596	67.5287054
HG03709	PJL	BHIL_MP02	22.1949841	72.9546922
HG03762	PJL	Kurumba_43_R1	11.3461788	76.668101
HG03765	PJL	BRAH_UP3M	26.519193	82.676502
HG03767	PJL	HGDP00262Pathan	32.6799373	70.3462383
HG03713	ITU	JEWS_KE05	10.4631596	76.1849113
HG03714	ITU	JEWS_KE05	10.7494978	76.1307904
HG03716	ITU	Vaish_132_R1	25.5428255	81.9031914

Sample id	Population	Genetically closest reference individual (from Moorjani et al. 2013 and HGDP)	GPS predicted Latitude	GPS predicted Longitude
HG03717	ITU	Vysya_155_R2	14.68	77.65
HG03718	ITU	Vysya_155_R2	14.68	77.65
HG03720	ITU	KURU_KE01	12.5840562	75.8100067
HG03722	ITU	Bhil_94_R1	22.0302958	73.0479749
HG03727	ITU	SHER_WB01	26.8764917	87.6973486
HG03729	ITU	Hallaki_143_R2	13.5884408	75.0263517
HG03730	ITU	MADI_AP1F	17.4602885	78.8752236
HG03731	ITU	KVGO_TN01	11.5856745	77.5210428
HG03742	ITU	KURU_KE03	12.2259467	75.5586224
HG03770	ITU	KALL_TN02	12.6400616	77.8631383
HG03771	ITU	KURU_KE05	12.4226855	75.8520276
HG03772	ITU	Vysya_153_R2	14.68	77.65
HG03773	ITU	Vysya_153_R2	14.68	77.65
HG03774	ITU	KVGO_TN03	12.0845333	77.5132611
HG03775	ITU	Hallaki_145_R2	14.3436943	74.3666337
HG03777	ITU	KVGO_TN05	11.5270228	77.6557275
HG03778	ITU	Naidu_180_R2	13.7796573	78.2170462
HG03779	ITU	Naidu_177_R2	13.8090751	78.5204843
HG03780	ITU	KVGO_TN02	11.9326843	77.4580764
HG03781	ITU	Lodi_203_R2	26.4003205	82.7747629
HG03782	ITU	KVGO_TN02	11.7474792	77.6140332
HG03784	ITU	Vysya_153_R2	14.68	77.65
HG03785	ITU	Vysya_153_R2	14.68	77.65
HG03786	ITU	Vysya_148_R2	14.68	77.65
HG03787	ITU	Bhil_94_R1	22.4129202	72.9290391
HG03788	ITU	BHIL_MP01	22.4111	73.0136824
HG03789	ITU	JEWS_KE05	11.0759652	76.3402512
HG03790	ITU	Velama_181_R2	16.1223659	75.2553097
HG03792	ITU	KVGO_TN03	11.9447059	77.39473
HG03861	ITU	Vysya_146_R2	14.68	77.65
HG03862	ITU	KVGO_TN03	11.9609336	77.4305174
HG03863	ITU	Hallaki_145_R2	14.3589945	74.4205592
HG03864	ITU	Velama_181_R2	16.1281408	75.548968
HG03866	ITU	KVGO_TN05	12.1325422	77.4774311
HG03867	ITU	BHIL_GU01	22.3740597	73.0508262
HG03868	ITU	KVGO_TN02	11.9692738	77.8483583
HG03869	ITU	BHIL_MP02	22.2894806	72.967991
HG03870	ITU	Hallaki_143_R2	14.3108616	74.3829447
HG03871	ITU	Velama_181_R2	16.0602436	75.6280244
HG03872	ITU	BHIL_MP01	22.9691516	72.7627116
HG03873	ITU	KALL_TN04	11.4800239	78.2330012

Sample id	Population	Genetically closest reference individual (from Moorjani et al. 2013 and HGDP)	GPS predicted Latitude	GPS predicted Longitude
HG03874	ITU	BHIL_MP01	22.4158487	73.335098
HG03875	ITU	JEWS_KE02	10.8017062	76.0951052
HG03882	ITU	Hallaki_143_R2	14.4863902	74.234862
HG03960	ITU	KALL_TN03	12.401105	77.6840105
HG03963	ITU	KALL_TN03	12.0491615	78.0459912
HG03965	ITU	KURU_KE03	11.9821604	75.9169819
HG03967	ITU	KVGO_TN01	12.1164019	77.2786812
HG03968	ITU	Velama_183_R2	16.5412828	75.7983515
HG03969	ITU	Velama_183_R2	16.4047257	75.8240831
HG03971	ITU	Hallaki_143_R2	13.7136398	74.9681828
HG03973	ITU	Lodi_203_R2	26.2327618	82.8149912
HG03974	ITU	Hallaki_143_R2	14.2041889	74.9102285
HG03976	ITU	KURU_KE04	12.3874597	75.7608083
HG03977	ITU	MADI_AP4M	17.5583384	79.1649722
HG03978	ITU	Kamsali_194_R2	14.9728278	77.4790162
HG04001	ITU	KURU_KE05	12.0342708	76.0419263
HG04002	ITU	KURU_KE04	12.9549597	75.3873498
HG04014	ITU	VEDD_SL03	8.92050172	80.0431883
HG04015	ITU	Naidu_176_R2	13.2825669	78.7000609
HG04017	ITU	Vysya_155_R2	14.68	77.65
HG04018	ITU	Hallaki_143_R2	14.4889921	74.2972676
HG04019	ITU	Velama_181_R2	16.891924	75.7870173
HG04020	ITU	Hallaki_140_R2	13.7794394	74.7902643
HG04022	ITU	KURU_KE03	12.412615	75.9806472
HG04023	ITU	Vysya_153_R2	14.68	77.65
HG04025	ITU	Vysya_155_R2	14.68	77.65
HG04026	ITU	Vysya_153_R2	14.68	77.65
HG04054	ITU	Vysya_153_R2	14.68	77.65
HG04056	ITU	Vysya_148_R2	14.68	77.65
HG04059	ITU	Naidu_180_R2	15.4276309	78.9408982
HG04060	ITU	JEWS_KE05	11.503674	76.5704318
HG04061	ITU	Madiga_164_R2	17.7655102	79.2944104
HG04062	ITU	MADI_AP1M	17.711694	79.2389862
HG04063	ITU	Vysya_146_R2	14.68	77.65
HG04070	ITU	KURU_KE04	12.6019566	75.9396504
HG04076	ITU	Hallaki_145_R2	13.6130851	74.8582779
HG04080	ITU	JEWS_KE05	10.860522	76.1326499
HG04090	ITU	Naidu_178_R2	13.5670943	78.6154922
HG04093	ITU	Hallaki_145_R2	14.2933818	74.4614135
HG04094	ITU	KURU_KE05	12.9353669	75.4368414
HG04096	ITU	Vysya_148_R2	14.68	77.65

Sample id	Population	Genetically closest reference individual (from Moorjani et al. 2013 and HGDP)	GPS predicted Latitude	GPS predicted Longitude
HG04098	ITU	Velama_183_R2	15.9700604	75.9687122
HG04118	ITU	Kurumba_45_R1	10.9207245	76.5547851
HG04198	ITU	Hallaki_143_R2	14.3841648	74.6857811
HG04200	ITU	BHIL_MP02	22.4041847	72.9261745
HG04202	ITU	KALL_TN03	11.9546807	77.9809486
HG04206	ITU	BHIL_MP02	22.4579641	72.8849898
HG04209	ITU	Naidu_177_R2	13.8657011	78.8314763
HG04211	ITU	KVGO_TN01	12.1460469	76.8750666
HG04212	ITU	KVGO_TN02	11.5389759	77.6408722
HG04214	ITU	BHIL_MP02	22.412742	72.9161265
HG04216	ITU	BHIL_MP01	22.9478804	73.3419329
HG04219	ITU	BHIL_MP01	22.9001561	72.9618559
HG04222	ITU	KALL_TN01	12.6684184	78.3146545
HG04225	ITU	KVGO_TN01	12.1564238	77.0200739
HG04235	ITU	SHER_WB01	26.7714798	87.6453194
HG04238	ITU	KURU_KE04	12.6158633	75.8089467
HG04239	ITU	Naidu_176_R2	13.7041787	78.8559799
HG03642	STU	Naidu_176_R2	13.4231714	78.7050098
HG03643	STU	MAKV_TN05	9.01322514	77.5492413
HG03644	STU	Velama_183_R2	15.7973152	75.9261923
HG03645	STU	Satnami_208_R2	19.9635761	85.0830464
HG03646	STU	Naidu_178_R2	13.9106142	78.8057598
HG03672	STU	BHIL_MP05	22.5112173	73.2539038
HG03673	STU	Kurumba_43_R1	10.9718967	76.7876398
HG03679	STU	Kurumba_45_R1	11.3825538	76.6403303
HG03680	STU	Naidu_177_R2	13.638009	78.5702784
HG03681	STU	Naidu_177_R2	13.8599027	78.6125067
HG03684	STU	MALA_AP3M	17.3277267	77.7138845
HG03685	STU	Naidu_177_R2	14.1716191	78.9498706
HG03686	STU	KALL_TN02	12.2082004	78.2984622
HG03687	STU	Naidu_176_R2	13.6980298	78.6895282
HG03689	STU	Lodi_204_R2	26.2294199	83.1877656
HG03690	STU	JEWS_KE04	10.2437743	77.1663529
HG03691	STU	KALL_TN04	11.5616463	78.1562519
HG03692	STU	KALL_TN01	12.059581	77.950103
HG03693	STU	KALL_TN04	11.696438	78.0079083
HG03694	STU	JEWS_KE05	11.4773564	75.8943725
HG03695	STU	KALL_TN04	11.5071567	77.7204169
HG03696	STU	Kurumba_44_R1	11.3314859	76.4772443
HG03697	STU	Naidu_178_R2	13.6974586	78.6622417
HG03698	STU	Naidu_178_R2	13.9027166	78.7248362

Sample id	Population	Genetically closest reference individual (from Moorjani et al. 2013 and HGDP)	GPS predicted Latitude	GPS predicted Longitude
HG03711	STU	Kurumba_45_R1	11.3865372	76.8002264
HG03733	STU	KALL_TN05	12.6083361	78.0021898
HG03736	STU	Kurumba_43_R1	11.1807645	76.7103483
HG03738	STU	KVGO_TN05	12.0960248	77.6822164
HG03740	STU	KALL_TN04	11.3979939	78.0905974
HG03741	STU	MAKV_TN04	8.65697019	77.6619486
HG03743	STU	Mala_188_R2	16.517642	78.1666232
HG03744	STU	Kurumba_45_R1	11.3232695	76.9445942
HG03745	STU	Naidu_177_R2	13.6996308	78.6644524
HG03746	STU	HGDP00070Balochi	29.8200944	67.7640309
HG03750	STU	JEWS_KE04	9.52470453	77.0263613
HG03752	STU	BHIL_MP05	22.1372316	73.1524392
HG03753	STU	KURU_KE02	12.2124034	75.8635538
HG03754	STU	JEWS_KE04	10.0101284	76.3190642
HG03755	STU	Naidu_177_R2	13.6707953	78.6228419
HG03756	STU	KVGO_TN01	11.6543549	77.1089564
HG03757	STU	Bhil_100_R2	22.3726251	73.0033938
HG03760	STU	KALL_TN04	11.1684799	77.8628966
HG03836	STU	JEWS_KE04	10.9083595	76.6294737
HG03837	STU	BHIL_MP05	22.2398486	73.0323736
HG03838	STU	KALL_TN04	11.8538412	78.1177141
HG03844	STU	MAKV_TN05	9.09972917	77.8053058
HG03846	STU	KALL_TN04	12.1291367	78.1170854
HG03848	STU	Naidu_177_R2	13.7837977	78.737894
HG03849	STU	Naidu_176_R2	13.5733994	78.7971203
HG03850	STU	Kurumba_45_R1	10.8542756	76.9908734
HG03851	STU	Naidu_178_R2	13.5352212	78.4464119
HG03854	STU	Kurumba_45_R1	11.2487212	76.3221997
HG03856	STU	MALA_AP2M	16.2959007	78.3131055
HG03857	STU	KALL_TN03	12.3505483	77.873927
HG03858	STU	KALL_TN05	12.7371238	78.0008865
HG03884	STU	MAKV_TN05	9.05900064	77.6393096
HG03885	STU	Naidu_176_R2	13.4282707	78.7716306
HG03886	STU	JEWS_KE04	10.0310748	76.3114596
HG03887	STU	Naidu_178_R2	13.5896211	78.6752934
HG03888	STU	JAIN_GU02	22.3700446	72.4993828
HG03890	STU	Naidu_178_R2	13.65719	78.3294557
HG03894	STU	KALL_TN01	12.2662258	78.0113769
HG03895	STU	KALL_TN03	12.6806063	77.7689108
HG03896	STU	KALL_TN04	11.6257935	78.4839192
HG03897	STU	Naidu_178_R2	13.6524143	78.546304

Sample id	Population	Genetically closest reference individual (from Moorjani et al. 2013 and HGDP)	GPS predicted Latitude	GPS predicted Longitude
HG03898	STU	MAKV_TN04	8.72381111	77.6605709
HG03899	STU	KALL_TN01	12.4328881	77.678726
HG03900	STU	KVGO_TN01	11.414127	76.9755357
HG03943	STU	MAKV_TN04	8.99994395	77.6639215
HG03944	STU	JEWS_KE04	10.5966844	76.5719695
HG03945	STU	Naidu_178_R2	14.0153865	78.209754
HG03947	STU	KALL_TN04	12.4921734	77.9391041
HG03949	STU	JEWS_KE04	10.670298	76.8563686
HG03950	STU	KURU_KE02	12.1362276	75.8266463
HG03951	STU	Naidu_178_R2	13.8160694	78.4597227
HG03953	STU	BHIL_MP01	22.5385468	72.9357648
HG03955	STU	KURU_KE04	12.3829171	75.7630653
HG03985	STU	Vysya_152_R2	15.2290447	77.3952979
HG03986	STU	Naidu_178_R2	13.8724802	78.6134638
HG03989	STU	JEWS_KE04	10.2188285	76.4623882
HG03990	STU	KURU_KE01	11.8930501	76.2142806
HG03991	STU	KURU_KE04	12.7837482	75.9398548
HG03995	STU	Naidu_176_R2	13.2997788	78.7733165
HG03998	STU	JEWS_KE04	10.7005074	76.7580479
HG03999	STU	Naidu_177_R2	13.902528	78.1968488
HG04003	STU	JEWS_KE05	9.9834873	76.2790008
HG04006	STU	KALL_TN05	12.7288019	78.0555651
HG04029	STU	Kurumba_43_R1	11.1770612	76.9650264
HG04033	STU	KALL_TN01	13.1354561	78.5411011
HG04035	STU	Naidu_177_R2	13.7981389	78.3647864
HG04038	STU	KURU_KE04	12.0622779	76.0184156
HG04039	STU	JEWS_KE04	9.9054158	77.2140926
HG04042	STU	Naidu_178_R2	13.6551728	78.7793134
HG04047	STU	KALL_TN04	10.5573251	78.271766
HG04075	STU	Naidu_177_R2	13.9330166	78.8449232
HG04099	STU	Lodi_204_R2	25.7847125	83.0571263
HG04100	STU	KALL_TN01	12.7584965	78.0898962
HG04106	STU	KVGO_TN03	11.6237536	77.6352617
HG04107	STU	KVGO_TN01	11.3959476	77.3496787
HG04210	STU	Naidu_176_R2	13.4911488	78.682816
HG04227	STU	Satnami_209_R2	20.2062987	85.1413998
HG04229	STU	VEDD_SL03	9.1915712	79.7433654
HG03006	BEB	Lodi_205_R2	25.7384422	82.7032561
HG03007	BEB	GOND_CA01	21.230088	80.3398859
HG03009	BEB	Tharu_109_R2	27.5118308	79.332012
HG03012	BEB	Tharu_107_R1	27.2570122	79.7982918

Sample id	Population	Genetically closest reference individual (from Moorjani et al. 2013 and HGDP)	GPS predicted Latitude	GPS predicted Longitude
HG03585	BEB	Tharu_109_R2	27.0940395	78.8636764
HG03589	BEB	Lodi_205_R2	26.2043588	81.6301165
HG03593	BEB	GOND_CA01	22.6791002	80.0050069
HG03594	BEB	Tharu_109_R2	27.250966	79.2678729
HG03595	BEB	Tharu_109_R2	27.0533045	79.5835647
HG03598	BEB	Tharu_109_R2	26.7599824	79.0660591
HG03600	BEB	Lodi_204_R2	24.9315303	82.6572768
HG03603	BEB	Lodi_205_R2	24.7938212	82.738914
HG03604	BEB	Lodi_205_R2	25.0670925	82.2934659
HG03607	BEB	Lodi_205_R2	26.4930087	81.4211966
HG03611	BEB	Tharu_109_R2	27.4011971	78.9350495
HG03615	BEB	Tharu_109_R2	26.9026817	79.2484372
HG03616	BEB	Tharu_109_R2	27.0259801	79.3353876
HG03793	BEB	Tharu_105_R1	28.1667745	79.9110272
HG03796	BEB	Tharu_109_R2	25.8673905	79.772009
HG03800	BEB	Satnami_210_R2	19.92761	83.7226493
HG03802	BEB	Tharu_109_R2	27.6842947	79.4809729
HG03803	BEB	Tharu_107_R1	27.1131711	80.0651194
HG03805	BEB	Tharu_109_R2	27.6975926	79.244073
HG03808	BEB	Tharu_109_R2	27.4217755	79.3601219
HG03809	BEB	Tharu_109_R2	27.4942342	78.9450774
HG03812	BEB	Tharu_109_R2	26.7605519	78.9052509
HG03814	BEB	Tharu_109_R2	26.8708024	79.3420445
HG03815	BEB	Tharu_109_R2	27.2992306	79.0537802
HG03817	BEB	Lodi_205_R2	25.5844556	81.6805365
HG03821	BEB	Tharu_108_R1	26.8609154	79.6904453
HG03823	BEB	Lodi_205_R2	26.7746479	81.1109709
HG03824	BEB	Tharu_109_R2	26.9622225	79.623225
HG03826	BEB	GOND_CA01	22.960198	80.7931743
HG03829	BEB	Tharu_109_R2	26.6474689	79.0735037
HG03830	BEB	Tharu_109_R2	26.520206	79.6661425
HG03832	BEB	Lodi_205_R2	24.2602409	82.0270781
HG03833	BEB	Tharu_110_R2	26.845359	79.8704346
HG03902	BEB	Tharu_108_R1	27.2758414	79.0549277
HG03905	BEB	GOND_CA01	22.8246238	79.7220754
HG03907	BEB	Lodi_205_R2	26.1841579	81.2862491
HG03908	BEB	Lodi_205_R2	26.1352365	81.3309907
HG03910	BEB	Tharu_109_R2	27.3104671	79.2103449
HG03911	BEB	Tharu_107_R1	27.7773633	79.919962
HG03913	BEB	Tharu_105_R1	27.490111	79.4263388
HG03914	BEB	Tharu_109_R2	27.4415383	79.0179286

Sample id	Population	Genetically closest reference individual (from Moorjani et al. 2013 and HGDP)	GPS predicted Latitude	GPS predicted Longitude
HG03916	BEB	Tharu_109_R2	26.5984952	79.426436
HG03917	BEB	Lodi_205_R2	26.2663931	80.8238487
HG03919	BEB	Tharu_108_R1	28.182566	79.1561579
HG03920	BEB	Tharu_109_R2	27.4484736	79.3880737
HG03922	BEB	Tharu_109_R2	27.4895114	79.2505661
HG03925	BEB	Lodi_205_R2	25.5718144	80.8954829
HG03926	BEB	Tharu_109_R2	26.2203623	79.4989209
HG03928	BEB	Tharu_109_R2	26.9033401	79.1429779
HG03931	BEB	Tharu_109_R2	26.5217455	80.2617022
HG03934	BEB	Tharu_109_R2	27.4896348	79.0881171
HG03937	BEB	Lodi_205_R2	25.8719219	81.7060854
HG03940	BEB	Lodi_204_R2	25.4236066	82.8411716
HG03941	BEB	Lodi_204_R2	25.1371612	82.8366486
HG04131	BEB	Tharu_109_R2	26.6722317	79.0116933
HG04134	BEB	Tharu_109_R2	26.4026067	78.9100398
HG04140	BEB	Tharu_109_R2	26.5298457	79.7727039
HG04141	BEB	Tharu_109_R2	26.9789162	79.5156566
HG04144	BEB	Hallaki_139_R2	15.5558732	74.8840735
HG04146	BEB	Lodi_205_R2	25.043504	81.0597972
HG04152	BEB	Tharu_107_R1	26.5505803	80.1775204
HG04153	BEB	Tharu_109_R2	27.2408058	79.4822462
HG04155	BEB	Lodi_205_R2	25.1131233	81.9092747
HG04156	BEB	Tharu_107_R1	26.6904008	79.7302332
HG04158	BEB	Tharu_109_R2	26.8220332	79.6271091
HG04159	BEB	Tharu_105_R1	27.1515498	79.3877011
HG04161	BEB	Tharu_105_R1	26.5566086	80.1539344
HG04162	BEB	Chenchu_31_R1	20.3893673	80.300346
HG04164	BEB	Tharu_109_R2	27.0597023	79.3022953
HG04171	BEB	Tharu_109_R2	26.856377	79.7163599
HG04173	BEB	Tharu_109_R2	27.5275514	79.498122
HG04176	BEB	Lodi_205_R2	25.3326977	81.2146181
HG04177	BEB	Lodi_205_R2	25.3017179	81.4832941
HG04180	BEB	Tharu_105_R1	27.5119127	80.0090685
HG04182	BEB	Lodi_205_R2	25.2338072	81.3243004
HG04183	BEB	Tharu_107_R1	26.6512572	79.8962828
HG04185	BEB	Satnami_208_R2	20.4464275	84.6539921
HG04186	BEB	Chenchu_32_R1	19.3999443	79.3984575
HG04188	BEB	Lodi_205_R2	25.6376835	81.819953
HG04189	BEB	Lodi_205_R2	24.999572	82.2297971
HG04194	BEB	SHER_WB01	26.4507785	86.3296279
HG04195	BEB	Tharu_107_R1	26.6707495	79.7367783

a.

K	Cross-validation error
2	0.50209
3	0.48944
4	0.48318
5	0.47823
6	0.47487
7	0.47418
8	0.47363
9	0.47339
10	0.47315
11	0.47307
12	0.47309
13	0.47158
14	0.47159
15	0.47206
16	0.47190
17	0.47316
18	0.47316
19	0.47450
20	0.47548

b.

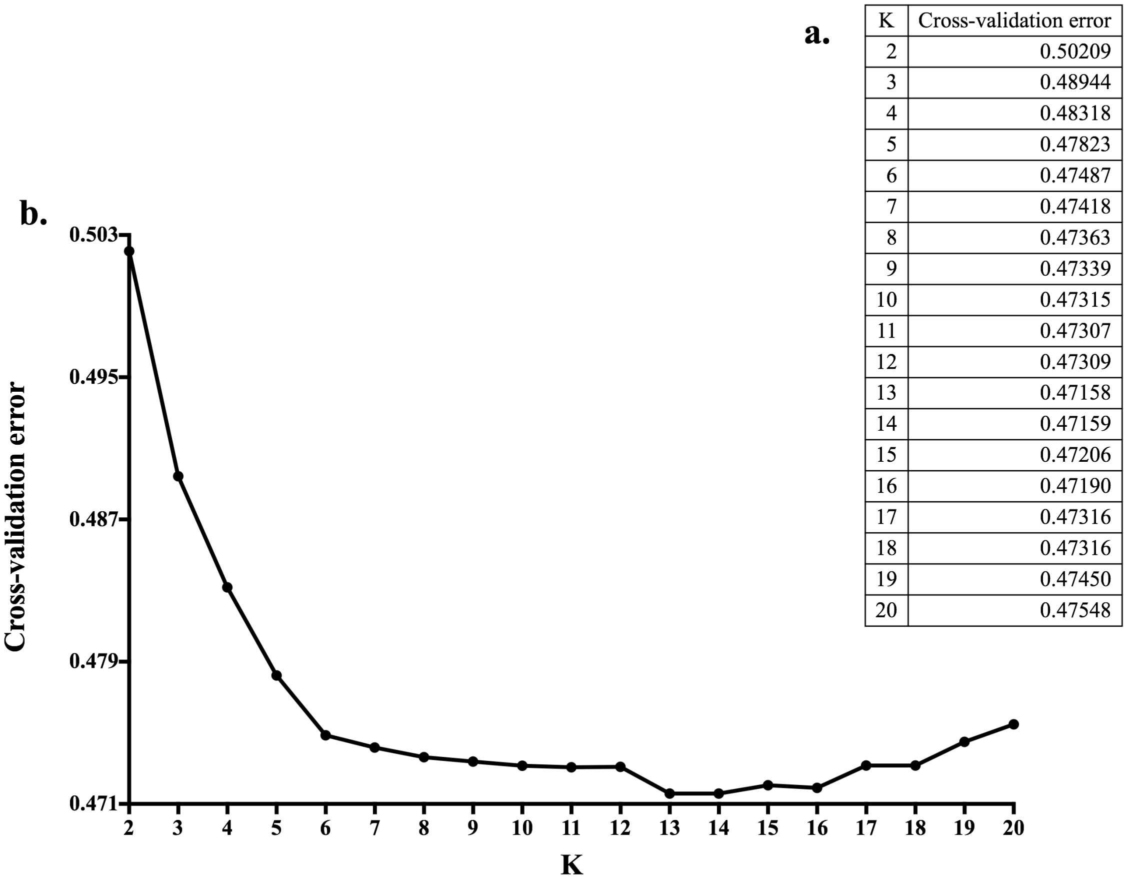

Figure S1. (a) Table showing proportion of Cross-Validation error (CVE) in ADMIXTURE carried out for the global dataset with different values of ancestral components (*K*) employed in the admixture analysis. The CVE was used to determine the optimum number of ancestral components (*K*) supported by the data. At *K*=13 the CVE was minimized. **(b)** Plot depicting the change of CVE with increasing number of ancestral components (*K*). The optimum number of ancestral components with lowest CVE was thirteen (*K*=13).

a.

K	Cross-validation error
2	0.49641
3	0.49392
4	0.49285
5	0.49257
6	0.49232
7	0.49216
8	0.49213
9	0.49393
10	0.49258
11	0.49290
12	0.49489

b.

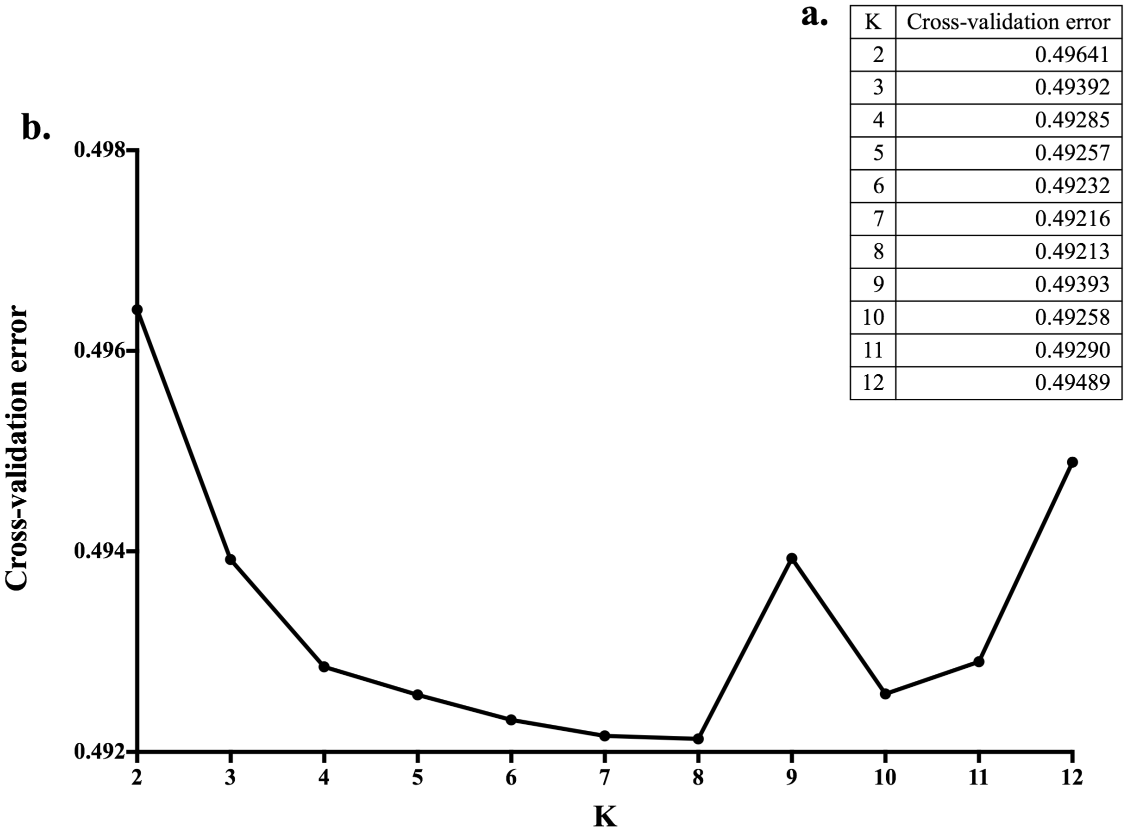

Figure S2. (a) Table showing proportion of Cross-Validation error (CVE) in ADMIXTURE carried out for the South Asian only dataset with different values of ancestral components (*K*) employed in the admixture analysis. The CVE was used to determine the optimum number of ancestral components (*K*) supported by the data. At *K*=8 the CVE was minimized. **(b)** Plot depicting the change of CVE with increasing number of ancestral components (*K*). The optimum number of ancestral components with lowest CVE was 8 (*K*=8).
